# Supplementary material for: Exposure to 16 h of normobaric hypoxia induces ionic edema in the healthy brain
Source: Nat Commun. 2021 Oct 13;12:5987. doi: 10.1038/s41467-021-26116-y (PMC8514510; doi:10.1038/s41467-021-26116-y)
Supplement: Supplementary file 1 — Supplementary Information [file 41467_2021_26116_MOESM1_ESM.pdf]

Supplementary Information

Exposure to 16 Hours of Normobaric Hypoxia induces Ionic Edema in the Healthy Brain

Supplementary Methods

Magnetic resonance imaging

Morphometry

Images were motion corrected and averaged [1] across multiple acquired volumetric T1-weighted images. Image post-processing steps further included removal of non-brain tissue using a hybrid watershed and surface deformation procedure [2], automated Talairach transformation, segmentation of subcortical white matter and deep gray matter volumetric structures [3, 4], intensity normalization [5], tessellation of the gray matter - white matter boundary and automated topology correction [6, 7]. To extract reliable volume estimates, images were uniformly post-processed with the longitudinal stream [8] in FreeSurfer. During the procedure an unbiased within-subject template space and image was created using robust, inverse consistent registration [1]. Several subsequent processing steps, such as skull stripping, Talairach transforms, atlas registration as well as spherical surface maps and parcellations relied on information from this within-subject template, significantly increasing reliability and statistical power [8].

Supplementary Figures

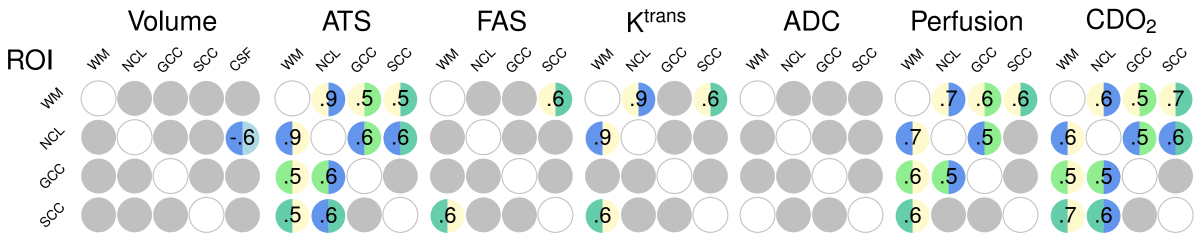

Supplementary Figure 1. Intra-modal correlations between changes in brain volume, ATS and FAS signals, K^{trans}, ADC values, perfusion and CDO_2 between all ROIs.
WM = white matter; NCL = nucleus lentiformis; GCC = genu corporis callosi; SCC = splenium corporis callosi; CSF = cerebrospinal fluid.

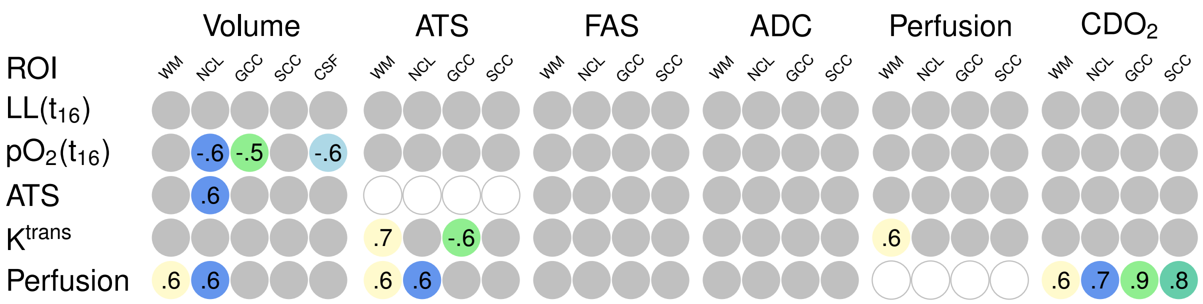

Supplementary Figure 2. Inter-modal correlations between changes in pO_2 (t_{16}), brain volume, ATS and FAS signals, K^{trans}, perfusion, and CDO_2 for each ROI. Naming conventions as in Supplementary Figure 1.

Supplementary Tables

Supplementary Table 1. Medication.

ID	Ibuprofen (dose and time point relative to the start of the experiment)	Motillium (dose and time point relative to the start of the experiment)
1	400 mg, 2 h 15 min; 400 mg, 9 h 15 min;	2 ml, 3 h 15 min
2	400 mg, 9 h	
3		
4		
5	400 mg, 4 h 40 min	
6	400 mg, 7 h	
7	400 mg, 5 h 45 min; 400 mg, 10 h 45 min; 400 mg, 14 h	
8	400 mg, 9 h 30 min	
9		
10		
11	400 mg, 8 h 45 min; 400 mg, 9 h 45 min	
12		
13	400 mg, 2 h 30 min; 600 mg, 6 h 15 min	2 ml, 1 h 15 min
14		
15		
16	400 mg, 5 h; 400 mg, 16 h	2 ml, 5 h; 2 ml, 7 h 30 min; 2 ml, 9 h 30 min; 2 ml, 16 h
17		
18		
19	400 mg, 5 h 45 min	
20	400 mg, 8 h	2 ml, 8 h
21		2 ml, 5 h 50 min
22		2 ml, 2 h 20 min
23	400 mg, 2 h 45 min	

Supplementary Table 2. Medication effects.

ROI	Measure	Estimates	Statistics
Genu Corporis Callosi	ADC	M1 = -1.27, M2 = -3.35, 95% CI (-3.64, 7.79)	t(19) = 0.76, p = 0.792, d = 0.17
Nucleus Lentiformis	ADC	M1 = 0.22, M2 = 1.01, 95% CI (-2.03, 0.45)	t(13) = -1.38, p = 0.768, d = -0.37
Splenium Corporis Callosi	ADC	M1 = -5.58, M2 = -6.18, 95% CI (-4.14, 5.35)	t(18) = 0.27, p = 0.792, d = 0.06

White Matter	ADC	M1 = 0.07, M2 = -0.35, 95% CI (-1.48, 2.32)	t(16) = 0.47, p = 0.792, d = 0.11
NA	AMS-C (t_{16})	M1 = 1.11, M2 = 1.64, 95% CI (-1.19, 0.14)	t(21) = -1.64, p = 0.116, d = -0.35
Genu Corporis Callosi	ATS	M1 = 2.59, M2 = 3.75, 95% CI (-4.68, 2.38)	t(20) = -0.68, p = 0.672, d = -0.15
Nucleus Lentiformis	ATS	M1 = 5.74, M2 = 7.66, 95% CI (-5.94, 2.1)	t(19) = -1, p = 0.66, d = -0.22
Splenium Corporis Callosi	ATS	M1 = 4.74, M2 = 5.5, 95% CI (-6.03, 4.52)	t(19) = -0.3, p = 0.768, d = -0.07
White Matter	ATS	M1 = 4.03, M2 = 6.09, 95% CI (-4.86, 0.74)	t(20) = -1.53, p = 0.564, d = -0.33
Genu Corporis Callosi	CDO_2	M1 = 15.81, M2 = -5.24, 95% CI (-8.68, 50.78)	t(18) = 1.49, p = 0.308, d = 0.34
Nucleus Lentiformis	CDO_2	M1 = -20.73, M2 = -31.4, 95% CI (-3.57, 24.91)	t(19) = 1.57, p = 0.308, d = 0.35
Splenium Corporis Callosi	CDO_2	M1 = -3.36, M2 = -11.07, 95% CI (-14.64, 30.06)	t(19) = 0.72, p = 0.479, d = 0.16
White Matter	CDO_2	M1 = -14.25, M2 = -21.27, 95% CI (-4.95, 18.98)	t(20) = 1.22, p = 0.313, d = 0.27
Genu Corporis Callosi	FAS	M1 = -4.54, M2 = -2.93, 95% CI (-13.61, 10.4)	t(20) = -0.28, p = 0.783, d = -0.06
Nucleus Lentiformis	FAS	M1 = -3.79, M2 = -5.74, 95% CI (-1.84, 5.72)	t(15) = 1.1, p = 0.684, d = 0.27
Splenium Corporis Callosi	FAS	M1 = -4.98, M2 = -3.61, 95% CI (-8.91, 6.18)	t(19) = -0.38, p = 0.783, d = -0.08
White Matter	FAS	M1 = -3.75, M2 = -6.35, 95% CI (-2.99, 8.2)	t(19) = 0.97, p = 0.684, d = 0.22
Genu Corporis Callosi	K^{trans}	M1 = -19.72, M2 = -15.65, 95% CI (-29.57, 21.44)	t(13) = -0.34, p = 0.736, d = -0.09
Nucleus Lentiformis	K^{trans}	M1 = -6.95, M2 = 8.62, 95% CI (-48.92, 17.78)	t(14) = -1, p = 0.445, d = -0.26
Splenium Corporis Callosi	K^{trans}	M1 = -4.64, M2 = 27.37, 95% CI (-85.63, 21.62)	t(15) = -1.27, p = 0.445, d = -0.32
White Matter	K^{trans}	M1 = 4.67, M2 = 26.74, 95% CI (-57.75, 13.6)	t(15) = -1.32, p = 0.445, d = -0.33
Genu Corporis Callosi	K^{trans} (Patlak)	M1 = 13.2, M2 = 60.25, 95% CI (-135.66, 41.57)	t(15) = -1.13, p = 0.994, d = -0.28
Nucleus Lentiformis	K^{trans} (Patlak)	M1 = 24.39, M2 = 14.3, 95% CI (-82.53, 102.7)	t(14) = 0.23, p = 0.994, d = 0.06
Splenium Corporis Callosi	K^{trans} (Patlak)	M1 = -3.45, M2 = 20.06, 95% CI (-99.06, 52.03)	t(13) = -0.67, p = 0.994, d = -0.18
White Matter	K^{trans} (Patlak)	M1 = -11.54, M2 = -11.69, 95% CI (-40.59, 40.9)	t(14) = 0.01, p = 0.994, d = 0
NA	LL-score (t_{16})	M1 = 5.64, M2 = 6.58, 95% CI (-2.74, 0.84)	t(21) = -1.1, p = 0.284, d = -0.23
Genu Corporis Callosi	Perfusion	M1 = 44.33, M2 = 19.61, 95% CI (-9.19, 58.62)	t(17) = 1.54, p = 0.568, d = 0.36
Nucleus Lentiformis	Perfusion	M1 = -2.36, M2 = -0.9, 95% CI (-22.94, 20.01)	t(19) = -0.14, p = 0.888, d = -0.03
Splenium Corporis Callosi	Perfusion	M1 = 19.85, M2 = 28.74, 95% CI (-36.82, 19.03)	t(19) = -0.67, p = 0.684, d = -0.15
White Matter	Perfusion	M1 = 6.04, M2 = 12.94, 95% CI (-23.95, 10.15)	t(20) = -0.84, p = 0.684, d = -0.18
NA	pO_2 (t_{16})	M1 = 36.7, M2 = 33.41, 95% CI (-0.4, 6.99)	t(21) = 1.85, p = 0.078, d = 0.4
Genu Corporis Callosi	Volume	M1 = 1.9, M2 = 2, 95% CI (-2.37, 2.18)	t(21) = -0.09, p = 0.931, d = -0.02
Nucleus Lentiformis	Volume	M1 = 0.3, M2 = 2.07, 95% CI (-3.75, 0.22)	t(21) = -1.85, p = 0.195, d = -0.39
Splenium Corporis Callosi	Volume	M1 = 1.14, M2 = 1.75, 95% CI (-3.93, 2.71)	t(21) = -0.38, p = 0.882, d = -0.08
Ventricles	Volume	M1 = -10.7, M2 = -14.53, 95% CI (-2.52, 10.18)	t(21) = 1.25, p = 0.372, d = 0.27
White Matter	Volume	M1 = 1.23, M2 = 2.54, 95% CI (-2.45, -0.18)	t(21) = -2.42, p = 0.125, d = -0.52

Statistics indicate paired t-tests, *p*-values are two-sided and were corrected according to Benjamini and Hochberg's procedure.

Supplementary Table 3. Hypoxia effects.

ROI	Measure	Estimates	Statistics
Genu Corporis Callosi	ADC	M = -2.46, 95% CI (-5.24, 0.33)	t(20) = -1.84, p = 0.108, d = -0.4
Nucleus Lentiformis	ADC	M = 0.69, 95% CI (0.07, 1.31)	t(14) = 2.38, p = 0.064, d = 0.61
Splenium Corporis Callosi	ADC	M = -5.91, 95% CI (-8.2, -3.62)	t(19) = -5.4, p = 0, d = -1.21
White Matter	ADC	M = -0.19, 95% CI (-1.09, 0.72)	t(17) = -0.44, p = 0.667, d = -0.1
Genu Corporis Callosi	ATS	M = 3.17, 95% CI (1.43, 4.91)	t(21) = 3.8, p = 0.001, d = 0.81
Nucleus Lentiformis	ATS	M = 6.66, 95% CI (4.66, 8.66)	t(20) = 6.94, p = 0, d = 1.52
Splenium Corporis Callosi	ATS	M = 5.1, 95% CI (2.54, 7.67)	t(20) = 4.15, p = 0, d = 0.91
White Matter	ATS	M = 5.06, 95% CI (3.62, 6.5)	t(21) = 7.31, p = 0, d = 1.56
Genu Corporis Callosi	CDO_2	M = 5.29, 95% CI (-9.99, 20.56)	t(19) = 0.72, p = 0.478, d = 0.16
Nucleus Lentiformis	CDO_2	M = -26.83, 95% CI (-34.1, -19.55)	t(20) = -7.69, p = 0, d = -1.68
Splenium Corporis Callosi	CDO_2	M = -7.4, 95% CI (-18.38, 3.59)	t(20) = -1.4, p = 0.235, d = -0.31
White Matter	CDO_2	M = -18.08, 95% CI (-24.09, -12.07)	t(21) = -6.26, p = 0, d = -1.33

Genu Corporis Callosi	FAS	M = -3.73, 95% CI (-9.58, 2.12)	t(21) = -1.33, p = 0.199, d = -0.28
Nucleus Lentiformis	FAS	M = -4.82, 95% CI (-6.71, -2.93)	t(16) = -5.42, p = 0, d = -1.31
Splenium Corporis Callosi	FAS	M = -4.33, 95% CI (-8, -0.65)	t(20) = -2.46, p = 0.031, d = -0.54
White Matter	FAS	M = -5.11, 95% CI (-7.89, -2.33)	t(20) = -3.83, p = 0.002, d = -0.84
Genu Corporis Callosi	K^{trans}	M = -17.82, 95% CI (-30.05, -5.59)	t(14) = -3.13, p = 0.028, d = -0.81
Nucleus Lentiformis	K^{trans}	M = 0.83, 95% CI (-15.74, 17.41)	t(15) = 0.11, p = 0.916, d = 0.03
Splenium Corporis Callosi	K^{trans}	M = 12.31, 95% CI (-14.82, 39.44)	t(16) = 0.96, p = 0.468, d = 0.23
White Matter	K^{trans}	M = 16.36, 95% CI (-1.76, 34.47)	t(16) = 1.91, p = 0.148, d = 0.46
Genu Corporis Callosi	K^{trans} (Patlak)	M = 38.11, 95% CI (-6.27, 82.48)	t(16) = 1.82, p = 0.348, d = 0.44
Nucleus Lentiformis	K^{trans} (Patlak)	M = 19.35, 95% CI (-25.2, 63.89)	t(15) = 0.93, p = 0.492, d = 0.23
Splenium Corporis Callosi	K^{trans} (Patlak)	M = 7.52, 95% CI (-29.15, 44.2)	t(14) = 0.44, p = 0.667, d = 0.11
White Matter	K^{trans} (Patlak)	M = -11.62, 95% CI (-31.18, 7.94)	t(15) = -1.27, p = 0.45, d = -0.32
Genu Corporis Callosi	Perfusion	M = 32.62, 95% CI (15.14, 50.1)	t(18) = 3.92, p = 0.002, d = 0.9
Nucleus Lentiformis	Perfusion	M = -1.52, 95% CI (-11.85, 8.8)	t(20) = -0.31, p = 0.761, d = -0.07
Splenium Corporis Callosi	Perfusion	M = 24.51, 95% CI (10.8, 38.21)	t(20) = 3.73, p = 0.002, d = 0.81
White Matter	Perfusion	M = 9.81, 95% CI (1.4, 18.21)	t(21) = 2.43, p = 0.032, d = 0.52
Genu Corporis Callosi	Volume	M = 1.95, 95% CI (0.84, 3.06)	t(22) = 3.65, p = 0.002, d = 0.76
Nucleus Lentiformis	Volume	M = 1.23, 95% CI (0.18, 2.27)	t(22) = 2.44, p = 0.029, d = 0.51
Splenium Corporis Callosi	Volume	M = 1.46, 95% CI (-0.17, 3.08)	t(22) = 1.86, p = 0.076, d = 0.39
Ventricles	Volume	M = -12.7, 95% CI (-15.9, -9.49)	t(22) = -8.22, p = 0, d = -1.71
White Matter	Volume	M = 1.91, 95% CI (1.29, 2.53)	t(22) = 6.37, p = 0, d = 1.33

Statistics indicate paired t-tests, p-values are two-sided and were corrected according to Benjamini and Hochberg's procedure.

Supplementary Table 4. Inter-ROI correlations.

ROI 1	ROI 2	Measure	Statistics
Genu Corporis Callosi	Nucleus Lentiformis	ADC	r = -0.19, 95% CI (-0.64, 0.36), p = 0.502
Genu Corporis Callosi	Splenium Corporis Callosi	ADC	r = 0.43, 95% CI (-0.01, 0.74), p = 0.168
Splenium Corporis Callosi	Nucleus Lentiformis	ADC	r = 0.27, 95% CI (-0.28, 0.69), p = 0.502
White Matter	Genu Corporis Callosi	ADC	r = 0.2, 95% CI (-0.3, 0.61), p = 0.502
White Matter	Nucleus Lentiformis	ADC	r = 0.52, 95% CI (0.01, 0.82), p = 0.168
White Matter	Splenium Corporis Callosi	ADC	r = 0.23, 95% CI (-0.28, 0.64), p = 0.502
Genu Corporis Callosi	Nucleus Lentiformis	ATS	r = 0.56, 95% CI (0.17, 0.8), p = 0.016
Genu Corporis Callosi	Splenium Corporis Callosi	ATS	r = 0.31, 95% CI (-0.14, 0.65), p = 0.177
Splenium Corporis Callosi	Nucleus Lentiformis	ATS	r = 0.6, 95% CI (0.22, 0.82), p = 0.012
White Matter	Genu Corporis Callosi	ATS	r = 0.51, 95% CI (0.12, 0.77), p = 0.017
White Matter	Nucleus Lentiformis	ATS	r = 0.86, 95% CI (0.68, 0.94), p < 0.001
White Matter	Splenium Corporis Callosi	ATS	r = 0.53, 95% CI (0.13, 0.78), p = 0.017
Genu Corporis Callosi	Nucleus Lentiformis	CDO_2	r = 0.49, 95% CI (0.05, 0.77), p = 0.038
Genu Corporis Callosi	Splenium Corporis Callosi	CDO_2	r = 0.17, 95% CI (-0.3, 0.57), p = 0.486
Splenium Corporis Callosi	Nucleus Lentiformis	CDO_2	r = 0.58, 95% CI (0.18, 0.81), p = 0.016
White Matter	Genu Corporis Callosi	CDO_2	r = 0.49, 95% CI (0.06, 0.77), p = 0.038
White Matter	Nucleus Lentiformis	CDO_2	r = 0.6, 95% CI (0.22, 0.82), p = 0.012
White Matter	Splenium Corporis Callosi	CDO_2	r = 0.67, 95% CI (0.34, 0.86), p = 0.006
Genu Corporis Callosi	Nucleus Lentiformis	FAS	r = 0.28, 95% CI (-0.23, 0.67), p = 0.329
Genu Corporis Callosi	Splenium Corporis Callosi	FAS	r = 0.37, 95% CI (-0.07, 0.69), p = 0.19
Splenium Corporis Callosi	Nucleus Lentiformis	FAS	r = 0.13, 95% CI (-0.39, 0.59), p = 0.64
White Matter	Genu Corporis Callosi	FAS	r = 0.4, 95% CI (-0.04, 0.71), p = 0.19
White Matter	Nucleus Lentiformis	FAS	r = 0.38, 95% CI (-0.12, 0.73), p = 0.196
White Matter	Splenium Corporis Callosi	FAS	r = 0.58, 95% CI (0.19, 0.82), p = 0.042

Genu Corporis Callosi	Nucleus Lentiformis	K^{trans}	r = 0.05, 95% CI (-0.48, 0.55), p = 0.864
Genu Corporis Callosi	Splenium Corporis Callosi	K^{trans}	r = 0.22, 95% CI (-0.33, 0.66), p = 0.521
Splenium Corporis Callosi	Nucleus Lentiformis	K^{trans}	r = 0.45, 95% CI (-0.06, 0.77), p = 0.16
White Matter	Genu Corporis Callosi	K^{trans}	r = 0.24, 95% CI (-0.31, 0.67), p = 0.521
White Matter	Nucleus Lentiformis	K^{trans}	r = 0.88, 95% CI (0.69, 0.96), p < 0.001
White Matter	Splenium Corporis Callosi	K^{trans}	r = 0.62, 95% CI (0.2, 0.85), p = 0.024
Genu Corporis Callosi	Nucleus Lentiformis	Perfusion	r = 0.54, 95% CI (0.09, 0.8), p = 0.033
Genu Corporis Callosi	Splenium Corporis Callosi	Perfusion	r = 0.31, 95% CI (-0.17, 0.67), p = 0.197
Splenium Corporis Callosi	Nucleus Lentiformis	Perfusion	r = 0.43, 95% CI (-0.02, 0.73), p = 0.073
White Matter	Genu Corporis Callosi	Perfusion	r = 0.6, 95% CI (0.21, 0.83), p = 0.012
White Matter	Nucleus Lentiformis	Perfusion	r = 0.67, 95% CI (0.33, 0.85), p = 0.006
White Matter	Splenium Corporis Callosi	Perfusion	r = 0.6, 95% CI (0.22, 0.82), p = 0.012
Genu Corporis Callosi	Nucleus Lentiformis	Volume	r = 0.3, 95% CI (-0.12, 0.64), p = 0.298
Genu Corporis Callosi	Splenium Corporis Callosi	Volume	r = 0.09, 95% CI (-0.33, 0.48), p = 0.758
Genu Corporis Callosi	Ventricles	Volume	r = -0.3, 95% CI (-0.63, 0.13), p = 0.298
Nucleus Lentiformis	Ventricles	Volume	r = -0.6, 95% CI (-0.81, -0.25), p = 0.03
Splenium Corporis Callosi	Nucleus Lentiformis	Volume	r = -0.23, 95% CI (-0.59, 0.2), p = 0.416
Splenium Corporis Callosi	Ventricles	Volume	r = -0.03, 95% CI (-0.44, 0.39), p = 0.888
White Matter	Genu Corporis Callosi	Volume	r = 0.1, 95% CI (-0.32, 0.49), p = 0.758
White Matter	Nucleus Lentiformis	Volume	r = 0.31, 95% CI (-0.12, 0.64), p = 0.298
White Matter	Splenium Corporis Callosi	Volume	r = 0.29, 95% CI (-0.14, 0.63), p = 0.298
White Matter	Ventricles	Volume	r = -0.45, 95% CI (-0.73, -0.05), p = 0.145

Statistics indicate Pearson correlations, p-values are two-sided and were corrected according to Benjamini and Hochberg's procedure.

Supplementary Table 5. Intra-ROI correlations between measures.

ROI	Measure 1	Measure 2	Statistics
Genu Corporis Callosi	ADC	AMS-C (t_{16})	r = -0.28, 95% CI (1.29, 2.53), p = 0.432
Nucleus Lentiformis	ADC	AMS-C (t_{16})	r = 0.39, 95% CI (15.14, 50.1), p = 0.432
Splenium Corporis Callosi	ADC	AMS-C (t_{16})	r = -0.01, 95% CI (-11.85, 8.8), p = 0.973
White Matter	ADC	AMS-C (t_{16})	r = -0.19, 95% CI (10.8, 38.21), p = 0.608
Genu Corporis Callosi	ADC	ATS	r = -0.37, 95% CI (1.43, 4.91), p = 0.428
Nucleus Lentiformis	ADC	ATS	r = -0.14, 95% CI (4.66, 8.66), p = 0.961
Splenium Corporis Callosi	ADC	ATS	r = -0.03, 95% CI (2.54, 7.67), p = 0.961
White Matter	ADC	ATS	r = 0.01, 95% CI (3.62, 6.5), p = 0.961
Genu Corporis Callosi	ADC	CDO_2	r = -0.03, 95% CI (-9.99, 20.56), p = 0.982
Nucleus Lentiformis	ADC	CDO_2	r = -0.01, 95% CI (-34.1, -19.55), p = 0.982
Splenium Corporis Callosi	ADC	CDO_2	r = -0.18, 95% CI (-18.38, 3.59), p = 0.982
White Matter	ADC	CDO_2	r = 0.09, 95% CI (-24.09, -12.07), p = 0.982
Genu Corporis Callosi	ADC	FAS	r = 0.45, 95% CI (-9.58, 2.12), p = 0.192
Nucleus Lentiformis	ADC	FAS	r = -0.05, 95% CI (-6.71, -2.93), p = 0.973
Splenium Corporis Callosi	ADC	FAS	r = -0.2, 95% CI (-8, -0.65), p = 0.83
White Matter	ADC	FAS	r = 0.01, 95% CI (-7.89, -2.33), p = 0.973
Genu Corporis Callosi	ADC	K^{trans}	r = 0.64, 95% CI (-30.05, -5.59), p = 0.076
Nucleus Lentiformis	ADC	K^{trans}	r = 0.62, 95% CI (-15.74, 17.41), p = 0.084
Splenium Corporis Callosi	ADC	K^{trans}	r = -0.24, 95% CI (-14.82, 39.44), p = 0.531
White Matter	ADC	K^{trans}	r = -0.02, 95% CI (-1.76, 34.47), p = 0.936
Genu Corporis Callosi	ADC	LL-score (t_{16})	r = -0.29, 95% CI (0.84, 3.06), p = 0.408
Nucleus Lentiformis	ADC	LL-score (t_{16})	r = 0.28, 95% CI (0.18, 2.27), p = 0.408

Splenium Corporis Callosi	ADC	LL-score (t_{16})	r = 0.06, 95% CI (-0.17, 3.08), p = 0.797
White Matter	ADC	LL-score (t_{16})	r = -0.28, 95% CI (-15.9, -9.49), p = 0.408
Genu Corporis Callosi	ADC	pO2 (t_{16})	r = 0.22, 95% CI (1.4, 18.21), p = 0.601
Nucleus Lentiformis	ADC	pO2 (t_{16})	r = -0.54, 95% CI (-5.24, 0.33), p = 0.156
Splenium Corporis Callosi	ADC	pO2 (t_{16})	r = -0.02, 95% CI (0.07, 1.31), p = 0.938
White Matter	ADC	pO2 (t_{16})	r = -0.19, 95% CI (-8.2, -3.62), p = 0.601
NA	AMS-C (t_{16})	pO2 (t_{16})	r = -0.54, 95% CI (10.8, 38.21), p = 0.007
Genu Corporis Callosi	ATS	AMS-C (t_{16})	r = 0.1, 95% CI (-24.09, -12.07), p = 0.838
Nucleus Lentiformis	ATS	AMS-C (t_{16})	r = -0.09, 95% CI (0.84, 3.06), p = 0.838
Splenium Corporis Callosi	ATS	AMS-C (t_{16})	r = 0.05, 95% CI (0.18, 2.27), p = 0.838
White Matter	ATS	AMS-C (t_{16})	r = 0.25, 95% CI (-0.17, 3.08), p = 0.838
Genu Corporis Callosi	ATS	CDO_2	r = -0.28, 95% CI (-7.89, -2.33), p = 0.445
Nucleus Lentiformis	ATS	CDO_2	r = 0.27, 95% CI (-30.05, -5.59), p = 0.445
Splenium Corporis Callosi	ATS	CDO_2	r = 0.23, 95% CI (-15.74, 17.41), p = 0.445
White Matter	ATS	CDO_2	r = -0.06, 95% CI (-14.82, 39.44), p = 0.812
Genu Corporis Callosi	ATS	FAS	r = 0.16, 95% CI (-1.09, 0.72), p = 0.63
Nucleus Lentiformis	ATS	FAS	r = 0.13, 95% CI (1.43, 4.91), p = 0.63
Splenium Corporis Callosi	ATS	FAS	r = -0.18, 95% CI (4.66, 8.66), p = 0.63
White Matter	ATS	FAS	r = -0.4, 95% CI (2.54, 7.67), p = 0.284
Genu Corporis Callosi	ATS	K^{trans}	r = -0.62, 95% CI (3.62, 6.5), p = 0.038
Nucleus Lentiformis	ATS	K^{trans}	r = 0.5, 95% CI (-9.58, 2.12), p = 0.093
Splenium Corporis Callosi	ATS	K^{trans}	r = 0.24, 95% CI (-6.71, -2.93), p = 0.391
White Matter	ATS	K^{trans}	r = 0.7, 95% CI (-8, -0.65), p = 0.012
Genu Corporis Callosi	ATS	LL-score (t_{16})	r = 0.11, 95% CI (-1.76, 34.47), p = 0.999
Nucleus Lentiformis	ATS	LL-score (t_{16})	r = -0.16, 95% CI (-9.99, 20.56), p = 0.999
Splenium Corporis Callosi	ATS	LL-score (t_{16})	r = -0.04, 95% CI (-34.1, -19.55), p = 0.999
White Matter	ATS	LL-score (t_{16})	r = 0, 95% CI (-18.38, 3.59), p = 0.999
Genu Corporis Callosi	ATS	pO2 (t_{16})	r = -0.24, 95% CI (-15.9, -9.49), p = 0.38
Nucleus Lentiformis	ATS	pO2 (t_{16})	r = -0.38, 95% CI (1.29, 2.53), p = 0.182
Splenium Corporis Callosi	ATS	pO2 (t_{16})	r = -0.12, 95% CI (15.14, 50.1), p = 0.597
White Matter	ATS	pO2 (t_{16})	r = -0.47, 95% CI (-11.85, 8.8), p = 0.104
Genu Corporis Callosi	CDO_2	AMS-C (t_{16})	r = 0.2, 95% CI (-6.71, -2.93), p = 0.399
Nucleus Lentiformis	CDO_2	AMS-C (t_{16})	r = -0.29, 95% CI (-8, -0.65), p = 0.272
Splenium Corporis Callosi	CDO_2	AMS-C (t_{16})	r = -0.34, 95% CI (-7.89, -2.33), p = 0.256
White Matter	CDO_2	AMS-C (t_{16})	r = -0.43, 95% CI (-30.05, -5.59), p = 0.184
Genu Corporis Callosi	CDO_2	LL-score (t_{16})	r = 0.09, 95% CI (4.66, 8.66), p = 0.716
Nucleus Lentiformis	CDO_2	LL-score (t_{16})	r = -0.41, 95% CI (2.54, 7.67), p = 0.18
Splenium Corporis Callosi	CDO_2	LL-score (t_{16})	r = -0.23, 95% CI (3.62, 6.5), p = 0.427
White Matter	CDO_2	LL-score (t_{16})	r = -0.37, 95% CI (-9.58, 2.12), p = 0.18
Genu Corporis Callosi	CDO_2	pO2 (t_{16})	r = -0.01, 95% CI (-15.74, 17.41), p = 0.95
Nucleus Lentiformis	CDO_2	pO2 (t_{16})	r = 0.42, 95% CI (-14.82, 39.44), p = 0.073
Splenium Corporis Callosi	CDO_2	pO2 (t_{16})	r = 0.43, 95% CI (-1.76, 34.47), p = 0.073
White Matter	CDO_2	pO2 (t_{16})	r = 0.44, 95% CI (-9.99, 20.56), p = 0.073
Genu Corporis Callosi	FAS	AMS-C (t_{16})	r = -0.17, 95% CI (-8, -0.65), p = 0.885
Nucleus Lentiformis	FAS	AMS-C (t_{16})	r = -0.11, 95% CI (-7.89, -2.33), p = 0.885
Splenium Corporis Callosi	FAS	AMS-C (t_{16})	r = 0.03, 95% CI (-30.05, -5.59), p = 0.895
White Matter	FAS	AMS-C (t_{16})	r = -0.46, 95% CI (-15.74, 17.41), p = 0.148
Genu Corporis Callosi	FAS	CDO_2	r = -0.03, 95% CI (-8.2, -3.62), p = 0.904
Nucleus Lentiformis	FAS	CDO_2	r = -0.08, 95% CI (-1.09, 0.72), p = 0.904
Splenium Corporis Callosi	FAS	CDO_2	r = 0.14, 95% CI (1.43, 4.91), p = 0.904

White Matter	FAS	CDO_2	r = 0.22, 95% CI (4.66, 8.66), p = 0.904
Genu Corporis Callosi	FAS	K^{trans}	r = 0.16, 95% CI (10.8, 38.21), p = 0.592
Nucleus Lentiformis	FAS	K^{trans}	r = 0.31, 95% CI (1.4, 18.21), p = 0.504
Splenium Corporis Callosi	FAS	K^{trans}	r = -0.35, 95% CI (-5.24, 0.33), p = 0.504
White Matter	FAS	K^{trans}	r = -0.25, 95% CI (0.07, 1.31), p = 0.504
Genu Corporis Callosi	FAS	LL-score (t_{16})	r = -0.25, 95% CI (2.54, 7.67), p = 0.536
Nucleus Lentiformis	FAS	LL-score (t_{16})	r = -0.14, 95% CI (3.62, 6.5), p = 0.807
Splenium Corporis Callosi	FAS	LL-score (t_{16})	r = 0.03, 95% CI (-9.58, 2.12), p = 0.898
White Matter	FAS	LL-score (t_{16})	r = -0.47, 95% CI (-6.71, -2.93), p = 0.132
Genu Corporis Callosi	FAS	pO2 (t_{16})	r = -0.05, 95% CI (-14.82, 39.44), p = 0.831
Nucleus Lentiformis	FAS	pO2 (t_{16})	r = 0.14, 95% CI (-1.76, 34.47), p = 0.792
Splenium Corporis Callosi	FAS	pO2 (t_{16})	r = 0.16, 95% CI (-9.99, 20.56), p = 0.792
White Matter	FAS	pO2 (t_{16})	r = 0.46, 95% CI (-34.1, -19.55), p = 0.136
Genu Corporis Callosi	K^{trans}	AMS-C (t_{16})	r = -0.33, 95% CI (-11.85, 8.8), p = 0.674
Nucleus Lentiformis	K^{trans}	AMS-C (t_{16})	r = 0.14, 95% CI (10.8, 38.21), p = 0.793
Splenium Corporis Callosi	K^{trans}	AMS-C (t_{16})	r = -0.25, 95% CI (1.4, 18.21), p = 0.674
White Matter	K^{trans}	AMS-C (t_{16})	r = -0.04, 95% CI (-5.24, 0.33), p = 0.891
Genu Corporis Callosi	K^{trans}	CDO_2	r = -0.33, 95% CI (-18.38, 3.59), p = 0.499
Nucleus Lentiformis	K^{trans}	CDO_2	r = 0.25, 95% CI (-24.09, -12.07), p = 0.499
Splenium Corporis Callosi	K^{trans}	CDO_2	r = 0.24, 95% CI (0.84, 3.06), p = 0.499
White Matter	K^{trans}	CDO_2	r = 0.17, 95% CI (0.18, 2.27), p = 0.506
Genu Corporis Callosi	K^{trans}	LL-score (t_{16})	r = -0.31, 95% CI (-0.17, 3.08), p = 0.53
Nucleus Lentiformis	K^{trans}	LL-score (t_{16})	r = 0.01, 95% CI (-15.9, -9.49), p = 0.969
Splenium Corporis Callosi	K^{trans}	LL-score (t_{16})	r = -0.32, 95% CI (1.29, 2.53), p = 0.53
White Matter	K^{trans}	LL-score (t_{16})	r = -0.21, 95% CI (15.14, 50.1), p = 0.555
Genu Corporis Callosi	K^{trans}	pO2 (t_{16})	r = 0.27, 95% CI (0.07, 1.31), p = 0.565
Nucleus Lentiformis	K^{trans}	pO2 (t_{16})	r = -0.16, 95% CI (-8.2, -3.62), p = 0.565
Splenium Corporis Callosi	K^{trans}	pO2 (t_{16})	r = 0.19, 95% CI (-1.09, 0.72), p = 0.565
White Matter	K^{trans}	pO2 (t_{16})	r = -0.27, 95% CI (1.43, 4.91), p = 0.565
NA	LL-score (t_{16})	AMS-C (t_{16})	r = 0.84, 95% CI (-34.1, -19.55), p = 0
NA	LL-score (t_{16})	pO2 (t_{16})	r = -0.39, 95% CI (-0.17, 3.08), p = 0.066
Genu Corporis Callosi	Perfusion	ADC	r = 0.08, 95% CI (0.07, 1.31), p = 0.746
Nucleus Lentiformis	Perfusion	ADC	r = 0.43, 95% CI (-8.2, -3.62), p = 0.572
Splenium Corporis Callosi	Perfusion	ADC	r = -0.16, 95% CI (-1.09, 0.72), p = 0.746
White Matter	Perfusion	ADC	r = 0.15, 95% CI (1.43, 4.91), p = 0.746
Genu Corporis Callosi	Perfusion	AMS-C (t_{16})	r = -0.01, 95% CI (15.14, 50.1), p = 0.964
Nucleus Lentiformis	Perfusion	AMS-C (t_{16})	r = 0.19, 95% CI (-11.85, 8.8), p = 0.964
Splenium Corporis Callosi	Perfusion	AMS-C (t_{16})	r = -0.05, 95% CI (10.8, 38.21), p = 0.964
White Matter	Perfusion	AMS-C (t_{16})	r = 0.1, 95% CI (1.4, 18.21), p = 0.964
Genu Corporis Callosi	Perfusion	ATS	r = -0.14, 95% CI (4.66, 8.66), p = 0.59
Nucleus Lentiformis	Perfusion	ATS	r = 0.62, 95% CI (2.54, 7.67), p = 0.012
Splenium Corporis Callosi	Perfusion	ATS	r = 0.3, 95% CI (3.62, 6.5), p = 0.276
White Matter	Perfusion	ATS	r = 0.58, 95% CI (-9.58, 2.12), p = 0.012
Genu Corporis Callosi	Perfusion	CDO_2	r = 0.91, 95% CI (-34.1, -19.55), p = 0
Nucleus Lentiformis	Perfusion	CDO_2	r = 0.7, 95% CI (-18.38, 3.59), p = 0
Splenium Corporis Callosi	Perfusion	CDO_2	r = 0.84, 95% CI (-24.09, -12.07), p = 0
White Matter	Perfusion	CDO_2	r = 0.57, 95% CI (0.84, 3.06), p = 0.006
Genu Corporis Callosi	Perfusion	FAS	r = 0, 95% CI (-6.71, -2.93), p = 0.995
Nucleus Lentiformis	Perfusion	FAS	r = -0.04, 95% CI (-8, -0.65), p = 0.995
Splenium Corporis Callosi	Perfusion	FAS	r = 0.08, 95% CI (-7.89, -2.33), p = 0.995

White Matter	Perfusion	FAS	r = -0.36, 95% CI (-30.05, -5.59), p = 0.456
Genu Corporis Callosi	Perfusion	K^{trans}	r = -0.22, 95% CI (-15.74, 17.41), p = 0.499
Nucleus Lentiformis	Perfusion	K^{trans}	r = 0.56, 95% CI (-14.82, 39.44), p = 0.056
Splenium Corporis Callosi	Perfusion	K^{trans}	r = 0.3, 95% CI (-1.76, 34.47), p = 0.348
White Matter	Perfusion	K^{trans}	r = 0.64, 95% CI (-9.99, 20.56), p = 0.024
Genu Corporis Callosi	Perfusion	LL-score (t_{16})	r = 0.03, 95% CI (0.18, 2.27), p = 0.97
Nucleus Lentiformis	Perfusion	LL-score (t_{16})	r = -0.04, 95% CI (-0.17, 3.08), p = 0.97
Splenium Corporis Callosi	Perfusion	LL-score (t_{16})	r = -0.01, 95% CI (-15.9, -9.49), p = 0.97
White Matter	Perfusion	LL-score (t_{16})	r = 0.02, 95% CI (1.29, 2.53), p = 0.97
Genu Corporis Callosi	Perfusion	pO2 (t_{16})	r = 0.07, 95% CI (-5.24, 0.33), p = 0.779
Nucleus Lentiformis	Perfusion	pO2 (t_{16})	r = -0.28, 95% CI (0.07, 1.31), p = 0.444
Splenium Corporis Callosi	Perfusion	pO2 (t_{16})	r = -0.08, 95% CI (-8.2, -3.62), p = 0.779
White Matter	Perfusion	pO2 (t_{16})	r = -0.39, 95% CI (-1.09, 0.72), p = 0.284
Genu Corporis Callosi	Volume	ADC	r = -0.39, 95% CI (1.29, 2.53), p = 0.226
Nucleus Lentiformis	Volume	ADC	r = 0.43, 95% CI (15.14, 50.1), p = 0.226
Splenium Corporis Callosi	Volume	ADC	r = -0.17, 95% CI (-11.85, 8.8), p = 0.637
White Matter	Volume	ADC	r = 0.07, 95% CI (10.8, 38.21), p = 0.793
Genu Corporis Callosi	Volume	AMS-C (t_{16})	r = 0.42, 95% CI (0.84, 3.06), p = 0.22
Nucleus Lentiformis	Volume	AMS-C (t_{16})	r = 0.19, 95% CI (0.18, 2.27), p = 0.599
Splenium Corporis Callosi	Volume	AMS-C (t_{16})	r = 0.13, 95% CI (-0.17, 3.08), p = 0.599
Ventricles	Volume	AMS-C (t_{16})	r = -0.25, 95% CI (-15.9, -9.49), p = 0.599
White Matter	Volume	AMS-C (t_{16})	r = 0.12, 95% CI (1.29, 2.53), p = 0.599
Genu Corporis Callosi	Volume	ATS	r = 0.22, 95% CI (1.4, 18.21), p = 0.335
Nucleus Lentiformis	Volume	ATS	r = 0.59, 95% CI (-5.24, 0.33), p = 0.02
Splenium Corporis Callosi	Volume	ATS	r = -0.48, 95% CI (0.07, 1.31), p = 0.054
White Matter	Volume	ATS	r = 0.37, 95% CI (-8.2, -3.62), p = 0.119
Genu Corporis Callosi	Volume	CDO_2	r = 0.23, 95% CI (-7.89, -2.33), p = 0.74
Nucleus Lentiformis	Volume	CDO_2	r = -0.07, 95% CI (-30.05, -5.59), p = 0.75
Splenium Corporis Callosi	Volume	CDO_2	r = -0.14, 95% CI (-15.74, 17.41), p = 0.74
White Matter	Volume	CDO_2	r = 0.17, 95% CI (-14.82, 39.44), p = 0.74
Genu Corporis Callosi	Volume	FAS	r = -0.1, 95% CI (-1.09, 0.72), p = 0.912
Nucleus Lentiformis	Volume	FAS	r = 0, 95% CI (1.43, 4.91), p = 0.996
Splenium Corporis Callosi	Volume	FAS	r = -0.17, 95% CI (4.66, 8.66), p = 0.912
White Matter	Volume	FAS	r = -0.09, 95% CI (2.54, 7.67), p = 0.912
Genu Corporis Callosi	Volume	K^{trans}	r = -0.17, 95% CI (3.62, 6.5), p = 0.575
Nucleus Lentiformis	Volume	K^{trans}	r = 0.52, 95% CI (-9.58, 2.12), p = 0.152
Splenium Corporis Callosi	Volume	K^{trans}	r = 0.15, 95% CI (-6.71, -2.93), p = 0.575
White Matter	Volume	K^{trans}	r = 0.33, 95% CI (-8, -0.65), p = 0.386
Genu Corporis Callosi	Volume	LL-score (t_{16})	r = 0.52, 95% CI (-1.76, 34.47), p = 0.055
Nucleus Lentiformis	Volume	LL-score (t_{16})	r = -0.06, 95% CI (-9.99, 20.56), p = 0.985
Splenium Corporis Callosi	Volume	LL-score (t_{16})	r = 0.23, 95% CI (-34.1, -19.55), p = 0.72
Ventricles	Volume	LL-score (t_{16})	r = 0, 95% CI (-18.38, 3.59), p = 0.989
White Matter	Volume	LL-score (t_{16})	r = -0.14, 95% CI (-24.09, -12.07), p = 0.853
Genu Corporis Callosi	Volume	Perfusion	r = 0.25, 95% CI (0.84, 3.06), p = 0.412
Nucleus Lentiformis	Volume	Perfusion	r = 0.35, 95% CI (0.18, 2.27), p = 0.23
Splenium Corporis Callosi	Volume	Perfusion	r = -0.06, 95% CI (-0.17, 3.08), p = 0.781
White Matter	Volume	Perfusion	r = 0.45, 95% CI (-15.9, -9.49), p = 0.148
Genu Corporis Callosi	Volume	pO2 (t_{16})	r = -0.51, 95% CI (15.14, 50.1), p = 0.02
Nucleus Lentiformis	Volume	pO2 (t_{16})	r = -0.56, 95% CI (-11.85, 8.8), p = 0.015
Splenium Corporis Callosi	Volume	pO2 (t_{16})	r = -0.14, 95% CI (10.8, 38.21), p = 0.536

Ventricles	Volume	pO2 (t_{16})	r = 0.57, 95% CI (1.4, 18.21), p = 0.015
White Matter	Volume	pO2 (t_{16})	r = -0.2, 95% CI (-5.24, 0.33), p = 0.439

Statistics indicate Pearson correlations, p-values are two-sided and were corrected according to Benjamini and Hochberg's procedure.

Supplementary Table 6. MR Sequence details.

	**Morpho-metry**	**Sodium mapping**		**Transfer constant K^{trans}**	**Diffusion imaging**	**Perfusion imaging**
		ATS	FAS			
Spatial resolution	1,0 x 1,0 x 1,0 mm^3	4,5 x 4,5 x 4,5 mm^3*	5,5 x 5,5 x 5,5 mm^3*	0,9 x 0,9 x 5,0 mm^3	1,6 x 1,6 x 3,0 mm^3	3,6 x 3,6 x 3,6 mm^3
Repetition time (TR)	1570 ms	160 ms	124 ms	5,28 ms	5100 ms	2500 ms
Echo time (TE)	2,63 ms	0,3 ms	0,3 ms	2,45 ms	72,8 ms	9,9 ms
Inversion time (TI_1)	900 ms		0,34 ms			700 ms
Inversion time (TI_2)						1800 ms
Acquisition time (TA)	4 min 8 s	10 min 40s	8 min 16 s	8 min 0 s	1 min 22 s	2 min 47 s
Projections		4000	4000			
Readout time (T_{RO})		20 ms	10 ms			
Temporal resolution				11,8 s		
B-values					0 s/mm^2<br>1000 s/mm^2	
Diffusion directions					12	
Tagged-control pairs						30

** nominal spatial resolution*

Supplementary References

1. Reuter M, Rosas HD, Fischl B. Highly Accurate Inverse Consistent Registration: A Robust Approach. *NeuroImage* **53**, 1181--1196 (2010).

2. Segonne F*, et al.* A hybrid approach to the skull stripping problem in MRI. *NeuroImage* **22**, 1060-1075 (2004).

3. Fischl B*, et al.* Whole brain segmentation: automated labeling of neuroanatomical structures in the human brain. *Neuron* **33**, 341-355 (2002).

4. Fischl B*, et al.* Sequence-independent segmentation of magnetic resonance images. *NeuroImage* **23**, S69-S84 (2004).

5. Sled JG, Zijdenbos AP, Evans AC. A nonparametric method for automatic correction of intensity nonuniformity in MRI data. *IEEE Trans Med Imaging* **17**, 87-97 (1998).

6. Fischl B, Liu A, Dale AM. Automated manifold surgery: constructing geometrically accurate

and topologically correct models of the human cerebral cortex. *IEEE Medical Imaging* **20**, 70-80 (2001).

7. Segonne F, Pacheco J, Fischl B. Geometrically accurate topology-correction of cortical surfaces using nonseparating loops. *IEEE Trans Med Imaging* **26**, 518-529 (2007).

8. Reuter M, Schmansky NJ, Rosas HD, Fischl B. Within-Subject Template Estimation for Unbiased Longitudinal Image Analysis. *NeuroImage* **61**, 1402--1418 (2012).
