# Supplementary material for: Exposure to 16 h of normobaric hypoxia induces ionic edema in the healthy brain
Source: Nat Commun. 2021 Oct 13;12:5987. doi: 10.1038/s41467-021-26116-y (PMC8514510; doi:10.1038/s41467-021-26116-y)
Supplement: Supplementary file 3 — Reporting Summary [file 41467_2021_26116_MOESM3_ESM.pdf]

nature research

Corresponding author(s): Armin Biller, Stephanie Badde

Last updated by author(s): Aug 2, 2021

Reporting Summary

Nature Research wishes to improve the reproducibility of the work that we publish. This form provides structure for consistency and transparency in reporting. For further information on Nature Research policies, see our Editorial Policies and the Editorial Policy Checklist.

Statistics

For all statistical analyses, confirm that the following items are present in the figure legend, table legend, main text, or Methods section.

n/a	Confirmed	
☐	☒	The exact sample size (*n*) for each experimental group/condition, given as a discrete number and unit of measurement
☐	☒	A statement on whether measurements were taken from distinct samples or whether the same sample was measured repeatedly
☐	☒	The statistical test(s) used AND whether they are one- or two-sided<br>*Only common tests should be described solely by name; describe more complex techniques in the Methods section.*
☐	☒	A description of all covariates tested
☐	☒	A description of any assumptions or corrections, such as tests of normality and adjustment for multiple comparisons
☐	☒	A full description of the statistical parameters including central tendency (e.g. means) or other basic estimates (e.g. regression coefficient) AND variation (e.g. standard deviation) or associated estimates of uncertainty (e.g. confidence intervals)
☐	☒	For null hypothesis testing, the test statistic (e.g. *F*, *t*, *r*) with confidence intervals, effect sizes, degrees of freedom and *P* value noted<br>*Give P values as exact values whenever suitable.*
☒	☐	For Bayesian analysis, information on the choice of priors and Markov chain Monte Carlo settings
☒	☐	For hierarchical and complex designs, identification of the appropriate level for tests and full reporting of outcomes
☐	☒	Estimates of effect sizes (e.g. Cohen's *d*, Pearson's *r*), indicating how they were calculated

Our web collection on statistics for biologists contains articles on many of the points above.

Software and code

Policy information about availability of computer code

Data collection	No software was used.

Data analysis	MR data were analyzed using<br>- FreeSurfer freesurfer-Darwin-lion-stable-pub-v5.3.0<br>-- recon-all version 1.379.2.73, part of FreeSurfer<br><br>- FSL version 5.0.10<br>-- sienax, part of FSL (version 5.0.10)<br>-- fslmaths, part of FSL (version 5.0.10)<br>-- asl_file, part of FSL (version 5.0.10)<br>-- fast, part of FSL (version 5.0.10)<br>-- oxford_asl, part of FSL (version 5.0.10)<br>-- FLIRT version 6.0, part of FSL<br>-- mcflirt, part of FSL (version 6.0)<br>-- BET version 2.1, part of FS<br><br>- 3D slicer (version 4.8.1)<br>-- PkModelling (version 4.10), 3D slicer module<br><br>- Horos (version 3.3.6)<br>-- IB DCE (version 20.11), Horos plugin<br><br>All statistical analysis were conducted using R (version 4.0.3)

For manuscripts utilizing custom algorithms or software that are central to the research but not yet described in published literature, software must be made available to editors and reviewers. We strongly encourage code deposition in a community repository (e.g. GitHub). See the Nature Research guidelines for submitting code & software for further information.

Data

Policy information about availability of data

All manuscripts must include a data availability statement. This statement should provide the following information, where applicable:

- Accession codes, unique identifiers, or web links for publicly available datasets
- A list of figures that have associated raw data
- A description of any restrictions on data availability

The data that have been generated in this study are available in the open science framework with the identifier DOI 10.17605/OSF.IO/V9H87[http://dx.doi.org/10.17605/OSF.IO/V9H87].
Figures 3 and 4 have associated raw data provided as source data file.

Field-specific reporting

Please select the one below that is the best fit for your research. If you are not sure, read the appropriate sections before making your selection.

☒ Life sciences ☐ Behavioural & social sciences ☐ Ecological, evolutionary & environmental sciences

For a reference copy of the document with all sections, see nature.com/documents/nr-reporting-summary-flat.pdf

Life sciences study design

All studies must disclose on these points even when the disclosure is negative.

Sample size	Sample size was determined based on power analyses assuming a medium effect size (GPower; Faul F, Erdfelder E, Buchner A, Lang A-G. Statistical power analyses using G*Power 3.1: Tests for correlation and regression analyses. Behavior Research Methods 41, 1149-1160 , 2009), and a beta of 0.8.
Data exclusions	Data exclusion was done separately for each statistical test, to-be-excluded outliers were defined as values smaller or larger than 3 standard deviations than the mean for t-tests and 2 standard deviations for correlation analyses, as the latter are more sensitive to outliers.
Replication	The experimental manipulation was replicated for each participant.
Randomization	Each participant was tested under the same experimental conditions, there were no groups in this study. The design relies on a within-participant comparison of values obtained before and after the experimental manipulation.
Blinding	There were no groups in this study, the design relies on a within-participant comparison of values obtained before and after the experimental manipulation. Therefore, blinding is not applicable, there is simply no information that could be subject to blinding.

Reporting for specific materials, systems and methods

We require information from authors about some types of materials, experimental systems and methods used in many studies. Here, indicate whether each material, system or method listed is relevant to your study. If you are not sure if a list item applies to your research, read the appropriate section before selecting a response.

Materials & experimental systems

n/a	Involved in the study
☒	☐ Antibodies
☒	☐ Eukaryotic cell lines
☒	☐ Palaeontology and archaeology
☒	☐ Animals and other organisms
☐	☒ Human research participants
☒	☐ Clinical data
☒	☐ Dual use research of concern

Methods

n/a	Involved in the study
☒	☐ ChIP-seq
☒	☐ Flow cytometry
☐	☒ MRI-based neuroimaging

Human research participants

Policy information about studies involving human research participants

Population characteristics	23 healthy, non-smoking, non-anaemic individuals (1 female; age: 25 ± 3 years; body mass index 22.6 ± 1.6 kg/m2; height: 182 ± 6 cm)
Recruitment	Posters at the University and in sports facilities in the community. This resulted in a very homogeneous sample of mostly young men. As outlined in our Discussion, we refrain from analyzing any predictors of altitude mountain sickness due to the homogeneity of the sample.
Ethics oversight	Ethics Committee of the Medical Department of the University of Heidelberg

Note that full information on the approval of the study protocol must also be provided in the manuscript.

Magnetic resonance imaging

Experimental design

Design type	pre- and post MR scans with artificially induced normobaric hypoxia in between
Design specifications	all participants were exposed to 16 hours under hypoxic conditions, MR scans were obtained before and after the exposure; there were no trials or blocks in this experiment.
Behavioral performance measures	No behavioral performance measures were obtained.

Acquisition

Imaging type(s)	structural, diffusion, perfusion, sodium, DCE
Field strength	3T

Sequence & imaging parameters

STRUCTURAL
- sequence type: tfl (single-shot TutboFLASH sequence)
- imaging type (FLASH)
- Fov 256 mm
- matrix size 256
- slice thickness 1 mm
- orientation sagittal
- TR / TE / flip angle 1570 ms / 2.63 ms / 9 dg

PERFUSION
- sequence type: ep2d_pasl (single-shot FID EPI sequence)
- pulsed arterial spin labelling (PICORE Q2TIPS)
- imaging type (EPI)
- Fov 230 mm
- matrix size 64
- slice thickness 3.6 mm
- orientation axial
- TR / TE / flip angle 2500 ms / 9.9 ms / 90 dg

DCE
- sequence type: fl3d_vibe (gradient echo)
- imaging type (GE)
- Fov 256 mm
- matrix size 256
- slice thickness 5 mm
- orientation axial

- TR / TE / flip angle 5.28 ms / 2.45 ms / 10 dg

SODIUM
ATS
- sequence type: density-adapted 3D radial projection reconstruction pulse sequence
- imaging type (Radial)
- Fov 286 mm
- matrix size 64
- slice thickness 4.5 mm
- orientation n.a.
- TR / TE / flip angle 160 ms / 0.3 ms / 0 dg

FAS
- sequence type: density-adapted 3D radial projection reconstruction pulse sequence
- imaging type (Radial)
- Fov 286 mm
- matrix size 52
- slice thickness 5.5 mm
- orientation n.a.
- TR / TE / flip angle 160 ms / 0.3 ms / 0 dg

Area of acquisition: Whole brain

Diffusion MRI ☒ Used ☐ Not used

Parameters
- sequence type: ep2d_diff (spin-echo EPI sequence)
- imaging type (EPI)
- Fov 210 mm
- matrix size 128
- slice thickness 3 mm
- orientation axial
- TR / TE / flip angle 5100 ms / 72.8 ms / -- dg

Preprocessing

Preprocessing software:
FMRIB'S Software Library FSL (version 5.0.10)
FreeSurfer (version 5.3.0)
Tofts --> pkModelling (version 4.8 as module in 3DSlicer version 4.8.1)
Patlak --> IB DCE (version 20.11)

Normalization: Sodium data of brain parenchyma was normalized to CSF signals. Spatial transformation: Physiologic brain data was linearly registered to anatomical data with 12 degrees of freedom (flirt, Part of FSL).

Normalization template: Talaraich template (as part of the longitudinal stream of FreeSurfer)

Noise and artifact removal: Hamming filter

Volume censoring: For most of the analyses no motion correction was necessary; for ASL-Perfusion data mcflirt of FSL

Statistical modeling & inference

Model type and settings: Not applicable because the listed types of models refer to fMRI studies or behavioral measures neither of which was obtained in the current study. See 'Effects tested' for information on the statistical analysis.

Effect(s) tested: The difference between pre- and post-exposure measurements normalized by pre-exposure values was tested against zero using t-tests and correlated across ROIs and measures using Pearson's correlation coefficient.

Specify type of analysis: ☐ Whole brain ☒ ROI-based ☐ Both

Anatomical location(s): FreeSurfer parcellations

Statistic type for inference (See Eklund et al. 2016): All inferences were based on ROI-based statistics, i.e., mean values across all voxels within a ROI

Correction: Benjamini & Hochberg FDR correction

Models & analysis

n/a	Involved in the study	
☒	☐	Functional and/or effective connectivity
☒	☐	Graph analysis
☒	☐	Multivariate modeling or predictive analysis
